# Altered Brain Function in First-Episode and Recurrent Depression: A Resting-State Functional Magnetic Resonance Imaging Study

**DOI:** 10.3389/fnins.2022.876121

**Published:** 2022-04-25

**Authors:** Jifei Sun, Limei Chen, Jiakai He, Zhongming Du, Yue Ma, Zhi Wang, Chunlei Guo, Yi Luo, Deqiang Gao, Yang Hong, Lei Zhang, Fengquan Xu, Jiudong Cao, Xiaobing Hou, Xue Xiao, Jing Tian, Jiliang Fang, Xue Yu

**Affiliations:** ^1^Guang’anmen Hospital, China Academy of Chinese Medical Sciences, Beijing, China; ^2^Graduate School, China Academy of Chinese Medical Sciences, Beijing, China; ^3^Dongzhimen Hospital, Beijing University of Chinese Medicine, Beijing, China; ^4^Institute of Acupuncture and Moxibustion, China Academy of Chinese Medical Sciences, Beijing, China; ^5^Beijing First Hospital of Integrated Chinese and Western Medicine, Beijing, China

**Keywords:** first depressive episode, recurrent depressive episodes, magnetic resonance imaging, regional homogeneity, amplitude of low-frequency fluctuation

## Abstract

**Background:**

Studies on differences in brain function activity between the first depressive episode (FDE) and recurrent depressive episodes (RDE) are scarce. In this study, we used regional homogeneity (ReHo) and amplitude of low-frequency fluctuations (ALFF) as indices of abnormal brain function activity. We aimed to determine the differences in these indices between patients with FDE and those with RDE, and to investigate the correlation between areas of abnormal brain function and clinical symptoms.

**Methods:**

A total of 29 patients with RDE, 28 patients with FDE, and 29 healthy controls (HCs) who underwent resting-state functional magnetic resonance imaging were included in this study. The ReHo and ALFF measurements were used for image analysis and further analysis of the correlation between different brain regions and clinical symptoms.

**Results:**

Analysis of variance showed significant differences among the three groups in ReHo and ALFF in the frontal, parietal, temporal, and occipital lobes. ReHo was higher in the right inferior frontal triangular gyrus and lower in the left inferior temporal gyrus in the RDE group than in the FDE group. Meanwhile, ALFF was higher in the right inferior frontal triangular gyrus, left anterior cingulate gyrus, orbital part of the left middle frontal gyrus, orbital part of the left superior frontal gyrus, and right angular gyrus, but was lower in the right lingual gyrus in the RDE group than in the FDE group. ReHo and ALFF were lower in the left angular gyrus in the RDE and FDE groups than in the HC group. Pearson correlation analysis showed a positive correlation between the ReHo and ALFF values in these abnormal areas in the frontal lobe and the severity of depressive symptoms (*P* < 0.05). Abnormal areas in the temporal and occipital lobes were negatively correlated with the severity of depressive symptoms (*P* < 0.05).

**Conclusion:**

The RDE and FDE groups had abnormal neural function activity in some of the same brain regions. ReHo and ALFF were more widely distributed in different brain regions and had more complex neuropathological mechanisms in the RDE group than in the FDE group, especially in the right inferior frontal triangular gyrus of the frontal lobe.

## Introduction

Major depressive disorder (MDD) is a common clinical psychiatric disorder characterized by affective, cognitive, and somatic symptoms. Most of its clinical manifestations are common symptoms such as depressed mood, diminished interest, slowed thinking, loss of appetite, and insomnia (Wolfers et al., 2015). The World Health Organization reports that an estimated 350 million people worldwide have depression and that depression is the leading cause of suicide, with approximately 800,000 people dying by suicide each year (Zhang et al., 2018). By 2030, depression is expected to become the disease with the largest burden worldwide (Friedrich, 2017). Currently, the diagnosis of MDD relies on clinical scale assessment of the patient and the experience of the psychiatrist; however, knowledge about precise neurobiological biomarkers is lacking.

According to the classification criteria of the 10th revision of the International Statistical Classification of Diseases and Related Health Problems, MDD is classified into the first depressive episode (FDE) and recurrent depressive episodes (RDE) (Yuksel et al., 2018). Previous studies have demonstrated differences in depressive and somatic symptoms (Roca et al., 2011), cognitive functioning (Roca et al., 2015; Zu et al., 2021; Varghese et al., 2022), and quality of life (Zu et al., 2021) between patients with RDE and those with FDE. Epidemiological surveys have shown that depression has a high recurrence rate and that, once the first episode has occurred, it relapses within 5 years. In addition, approximately 80% of patients with depression have a history of two recurrences (Burcusa and Iacono, 2007). Moreover, the severity of MDD increases with the number of relapses (Harlev et al., 2021), and it is increasingly being recognized that the challenge in patients with depression is preventing relapse rather than promoting recovery (Fava et al., 2017). Therefore, the differences in pathogenesis between RDE and FDE need to be elucidated from a neuropathological perspective.

In recent years, with rapid developments in neuroimaging technology, resting-state functional magnetic resonance imaging (rs-fMRI) has gradually been applied to the study of insomnia (Marques et al., 2018), schizophrenia (Sheffield and Barch, 2016), autism (Bathelt and Geurts, 2021), and other psychiatric systemic disorders. It has also been applied to the study of MDD subtypes (Brown et al., 2019; Yue et al., 2020). rs-fMRI indirectly reflects neuronal spontaneous activity by measuring the blood oxygen level-dependent (BOLD) signal, which enables the investigation of brain function abnormalities in the early disease stage and has advantages of simplicity, non-invasiveness, and reproducibility (Lee et al., 2013). Regional homogeneity (ReHo) and amplitude of low-frequency fluctuations (ALFF) are two of the commonly used metrics in rs-fMRI analysis. ReHo mainly assesses the synchronous reflection of the sequence between a given voxel and its neighboring voxels, reflecting the temporal homogeneity of signals related to regional blood oxygen levels (Zang et al., 2004). ALFF is the sum of the spectral amplitude of each voxel signal in the low-frequency range (usually 0.01–0.08 Hz), reflecting the amplitude of low-frequency fluctuations caused by spontaneous neuronal activity (Zang et al., 2007). Combining the ReHo and ALFF methods can contribute to a better understanding of the abnormalities of brain function in patients with MDD (Ni et al., 2016).

However, the combination of ALFF and ReHo has been mainly applied in studies on mild cognitive impairment (Ni et al., 2016), schizophrenia (Zhao et al., 2018), and anxiety disorders (Shen et al., 2020), whereas the differences between FDE and RDE have not been investigated. A study in patients with FDE and remitting MDD (rMDD) showed differences in ALFF and ReHo in the temporal lobes, although patients with rMDD had been cured with antidepressant medication (Yang et al., 2018). Another study observed alterations in bilateral frontal BOLD signals in patients with FDE or RDE; however, the study used a task-state observation approach (Yuksel et al., 2018). The frontal lobe is an important brain region in the pathogenesis of MDD, and previous studies have also found differences in frontal lobe function between FDE and RDE (Talarowska et al., 2015).

In this study, we aimed to determine the differences in brain function activity among patients with FDE, patients with RDE, and healthy controls (HCs) by using the ReHo and ALFF methods based on rs-fMRI techniques. Further, we also aimed to analyze the correlation between different brain areas and clinical characteristics. We hypothesized that differences would be observed in the brain neural circuits of patients with FDE and those with RDE, especially in areas closely related to the frontal lobe. This study will provide a neuroimaging basis for the differences in neuropathological mechanisms between FDE and RDE and some insights for clinical research.

## Materials and Methods

### Participants

A total of 59 outpatients with MDD from Guang’anmen Hospital, Chinese Academy of Traditional Chinese Medicine, Beijing First Hospital of Integrative Medicine, were recruited for this study. All patients met the Diagnostic and Statistical Manual of Mental Disorders Fifth Edition criteria for MDD. We used the 17-item Hamilton Rating Scale for Depression (HAMD-17) (Hamilton, 1960) to assess the severity of depression in all patients and classified these patients into those with RDE [*n* = 31; mean frequency of recurrence, 2.27 (standard deviation = 0.79)] and those with FDE (*n* = 28, 0 recurrence). The inclusion criteria were (1) age 18–55 years; (2) HAMD-17 score > 17; and (3) The FDE group all had their FDE prior to enrollment and were not receiving any antidepressant medication. The RDE group had a previous history of depression, cured by antidepressant medication, now recurring and a history of antidepressant withdrawal for at least 4 weeks prior to enrollment. We also included 29 sex- and age-matched HCs (21 women and 8 men) who (1) were aged 18–55 years, (2) had a HAMD–17 score of < 7, (3) had right-handedness, and (4) had no history of any mental illness in first-degree relatives.

The exclusion criteria for patients and HCs were as follows: (1) serious mental illness and other diseases such as cardiovascular and cerebrovascular disorders; (2) history of drug and alcohol abuse; (3) any contraindications to MRI, such as presence of a heart pacemaker, metal fixed false teeth, or severe claustrophobia; (4) pregnant or lactating status; and (5) bipolar disorder or suicidal ideation.

All patients were required to sign an informed consent form before enrollment. This study was approved by the ethics committee of Guang’anmen Hospital, Chinese Academy of Traditional Chinese Medicine.

### Scan Acquisition

All patients in this study underwent MRI using a Magnetom Skyra 3.0-T scanner (Siemens, Erlangen, Germany). Before the scanning procedure, the patients were instructed to remain awake and avoid active thinking. During the scanning process, the patients were required to wear earplugs and noise-canceling headphones, to use a hood to immobilize the head, and to lie flat on the examination bed. The scanning procedure involved a localizer scan, high-resolution three-dimensional T1-weighted imaging, and BOLD-fMRI.

The scanning parameters were as follows: for three-dimensional T1-weighted imaging, time repetition/time echo = 2,500/2.98 ms, flip angle = 7°, matrix = 64 × 64, field of view = 256 mm × 256 mm, slice thickness = 1 mm, slice number = 48, slices = 192, scanning time = 6 min 3 s; for BOLD-fMRI, time repetition/time echo = 2,000/30 ms, flip angle = 90°, matrix = 64 × 64, field of view = 240 mm × 240 mm, slice number = 43, slice thickness/spacing = 3.0/1.0 mm, number of obtained volumes = 200, and scanning time = 6 min 40 s.

### Image Processing

#### Functional Magnetic Resonance Imaging Data Preprocessing

The rs-fMRI data were preprocessed using DPARSF (Data Processing Assistant for rs-fMRI) software (DPABI5.0)^1^ (Yan and Zang, 2010) in MATLAB (Mathworks Inc., Natick, MA, United States), according to the following process: (1) conversion of DICOM raw data to NIFTI format; (2) removal of the first 10 time points to place the data in a stable state; (3) slice timing; (4) realignment of head motion (removal of patients with head movements > 2 mm in any direction and motor rotation > 2°); (5) regression of covariates, including brain white matter signal, cerebrospinal fluid signal, and head movement parameters; (6) spatial normalization (the functional images of all patients were converted to Montreal Neurological Institute standard space using the DARTEL method); and (7) linear detrending and filtering (0.01–0.08 Hz).

#### Regional Homogeneity Analysis

The DPARSF (Data Processing Assistant for Resting-State fMRI Advanced Edition_V5.1_201001, see text footnote 1) software was used to analyze the ReHo and ALFF of the pre-processed data. The similarity of the time series of each voxel to its neighboring voxels (26 neighboring voxels) was assessed using the Kendall’s coefficient of concordance (KCC) (Kendall, 1990), i.e., ReHo values. The whole-brain ReHo images of the subjects were obtained by calculating the KCC values of the whole-brain voxels. To improve the signal-to-noise ratio, the Re Ho images were spatially smoothed using a 6 mm × 6 mm × 6 mm full-width half-height Gaussian kernel.

#### Amplitude of Low-Frequency Fluctuations Analysis

Data were spatially normalized and smoothed, and a fast Fourier transform was performed to switch the time series to the frequency domain to obtain the power spectrum. The square root of the power spectrum at each frequency was calculated to obtain the average square root of the ALFF measurement for each voxel in the range of 0.01–0.08 Hz. Finally, time bandpass filtering (0.01–0.08 Hz) was performed. To reduce the inter-individual variability, ALFF was transformed to zALFF using Fisher’s *z* transformation before statistical analysis.

### Statistical Analyses

#### Clinical Data Analysis

Clinical data were analyzed using the SPSS 23.0 statistical software (IBM Corporation, Somers, NY, United States). One-way analysis of variance was used to compare age and educational level among the three groups, and the chi-square test was used to compare sex differences. A two-sample *t*-test was used to compare the duration of disease, HAMD-17 scores, and frequency of recurrence between the two patient groups, with a threshold of *P* < 0.05 (two-tailed) set as statistically significant.

#### Functional Magnetic Resonance Imaging Data Analysis

Imaging data were analyzed using the DPARSF toolbox, and a voxel-based one-way analysis of variance was performed to compare the whole-brain ReHo/ALFF map among the three groups. Sex, age, educational level, and framewise displacement (a metric derived from Jenkinson’s formula) were used as covariates, and brain areas with ReHo/ALFF differences among the three groups were corrected for Gaussian random fields. The corrected cluster level was set at *P* < 0.05 (two-tailed), and threshold voxel levels of *P* < 0.005 were defined as statistically different. We performed *post-hoc t*-test analysis using DPARSF 5.1 software for two-by-two comparisons between groups, and Bonferroni correction was applied to the results, setting a threshold of *P* < 0.016 (0.05/3) for statistical significance. The threshold was set to clusters > 10 voxels.

To verify the relationship between ReHo/ALFF values and clinical symptoms, we extracted the mean ReHo/ALFF values of three different brain regions and performed Pearson correlation analysis of the clinical scale scores of each group. Significance was set at a statistical threshold of *P* < 0.05 (two-tailed).

## Results

### Characteristics of Research Samples

Two patients with RDE were excluded because of excessive head movement displacement. Therefore, a total of 29 patients with RDE, 28 patients with FDE, and 29 HCs met the inclusion criteria. No statistical differences among the three groups were found in terms of sex, age, and years of education. The HAMD-17 scores were not statistically different between the RDE and FDE groups, whereas a statistical difference was observed in the duration of illness and frequency of recurrence (Table 1).

**TABLE 1:** Demographic and clinical characteristics of the study participants.

Variable	RDE (*n* = 29)	FDE (*n* = 28)	HCs (*n* = 29)	*t*(*F*)/χ^2^	*P* -value
Sex (M/F)	8/21	8/20	8/21	0.043	0.958^a^
Age (years)	33.62 ± 10.28	32.85 ± 9.77	33.24 ± 9.42	0.018	0.982^b^
Education (years)	14.96 ± 2.44	14.28 ± 2.80	14.93 ± 3.05	0.538	0.586^b^
Duration of illness (months)	22.96 ± 11.49	2.28 ± 0.93	NA	9.487	<0.001^c^*
HAMD-17 score	23.31 ± 3.26	23.10 ± 3.16	NA	0.238	0.812^c^
Frequency of recurrence	2.34 ± 0.76	0	NA	16.133	<0.001^c^*

*RDE, recurrent depressive episode; FDE, first depressive episode; HCs, healthy controls; HAMD-17, 17-item Hamilton Rating Scale for Depression; NA, not applicable.*

*^a^The P-values of sex distribution among the three groups were obtained using the chi-square test.*

*^b^P-value from one-way analysis of variance tests.*

*^c^P-value from a two-sample t-test.*

*Significant difference.

### Differences in Regional Homogeneity/Amplitude of Low-Frequency Fluctuations Between the Recurrent Depressive Episodes, First Depressive Episode, and Healthy Controls

**FIGURE 1:**
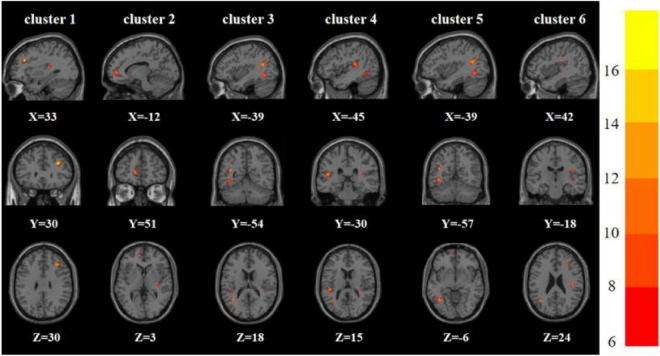
Brain regions with abnormal ReHo among the three groups based on one-way analysis of variance. The color bars indicate the *F*-value.

**FIGURE 2:**
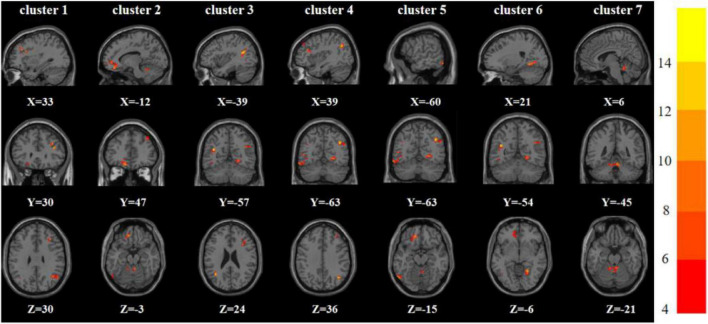
Brain regions with abnormal ALFF among the three groups based on one-way analysis of variance. The color bars indicate the *F*-value.

One-way analysis of variance showed significant differences in ReHo and ALFF among the three groups in the right inferior frontal triangular gyrus, left anterior cingulate cortex, left middle temporal gyrus/left angular gyrus, and left inferior temporal gyrus/left inferior occipital gyrus. Meanwhile, ReHo was significantly different in the left superior temporal gyrus and right Rolandic operculum gyrus. ALFF was also significantly different in the right angular gyrus, right lingual gyrus, and Vermis_3 (Tables 2, 3 and Figures 1, 2).

**TABLE 2:** ReHo differences in RDE, FDE, and HCs.

Clusters	Brain regions	MNI peak			Cluster size	*F/T*-value (peak)
		
		*X*	*Y*	*Z*		
**Differences among three groups**						
1	Right inferior frontal triangular gyrus	33	30	30	27	17.934^a^
2	Left anterior cingulate cortex	−12	51	3	25	12.561^a^
3	Left middle temporal gyrus	−39	−54	18	34	15.505^a^
	Left angular gyrus					
4	Left superior temporal gyrus	−45	−30	15	29	13.698^a^
5	Left inferior temporal gyrus	−39	−57	−6	53	12.601^a^
	Left inferior occipital gyrus					
6	Right Rolandic operculum gyrus	42	−18	24	34	11.938^a^
**RDE vs. FDE**						
1	Right inferior frontal triangular gyrus	33	30	30	19	4.344^b^
2	Left inferior temporal gyrus	−42	−57	−6	35	−4.270^b^
**RDE vs. HCs**						
1	Right inferior frontal triangular gyrus	33	30	27	23	4.745^b^
2	Left anterior cingulate cortex	−12	51	3	25	4.272^b^
3	Left superior temporal gyrus	−45	−30	15	29	4.543^b^
4	Left angular gyrus	−39	−63	27	17	−2.881^b^
**FDE vs. HCs**						
1	Left inferior temporal gyrus	−44	−63	−9	25	2.905^b^
2	Left middle temporal gyrus	−39	−51	15	23	−2.816^b^
	Left angular gyrus					

*MNI peak, coordinates of primary peak locations in the Montreal Neurological Institute space.*

*^a^F-value of the peak voxel showing gray matter volume differences among the three groups.*

*^b^T-value of the peak voxel showing gray matter volume differences among the three groups (post hoc two-group comparisons).*

**TABLE 3:** ALFF differences in RDE, FDE, and HCs.

Clusters	Brain regions	MNI Peak			Cluster size	*F/T*-value (peak)
		
		*X*	*Y*	*Z*		
**Differences among three groups**						
1	Right inferior frontal triangular gyrus	33	30	30	22	12.285^a^
2	Left anterior cingulate cortex	−12	42	−18	109	11.440^a^
	Orbital part of the left middle frontal gyrus					
3	Left angular gyrus	−39	−57	24	45	15.704^a^
	Left middle temporal gyrus					
4	Right angular gyrus	39	−63	36	36	14.556^a^
5	Left inferior temporal gyrus	−60	−63	−15	31	12.380^a^
	Left inferior occipital gyrus					
6	Right lingual gyrus	21	−54	−6	70	13.548^a^
7	Vermis_3	6	−45	−21	51	12.253^a^
**RDE vs. FDE**						
1	Right inferior frontal triangular gyrus	34	18	25	14	4.222^b^
2	Left anterior cingulate cortex	−6	36	6	14	3.376^b^
3	Orbital part of the left middle frontal gyrus	−12	48	−3	17	3.942^b^
4	Orbital part of the left superior frontal gyrus	−12	42	18	42	4.445*^b^*
5	Right angular gyrus	39	−63	33	15	4.167^b^
6	Right lingual gyrus	15	−51	−9	65	−2.909^b^
**RDE vs. HCs**						
1	Left anterior cingulate cortex	−15	48	−3	24	3.641^b^
2	Left angular gyrus	−39	−60	21	18	−3.094^b^
3	Right angular gyrus	42	−63	36	26	4.097^b^
**FDE vs. HCs**						
1	Left middle temporal gyrus	−42	−51	12	36	−3.015^b^
	Left angular gyrus					
2	Left inferior temporal gyrus	−60	−63	−15	26	4.055^b^
3	Vermis_3	6	−45	−21	15	3.830^b^

*MNI peak, coordinates of primary peak locations in the Montreal Neurological Institute space.*

*^a^F-value of the peak voxel showing gray matter volume differences among the three groups.*

*^b^T-value of the peak voxel showing gray matter volume differences among the three groups (post hoc two-group comparisons).*

**FIGURE 3:**
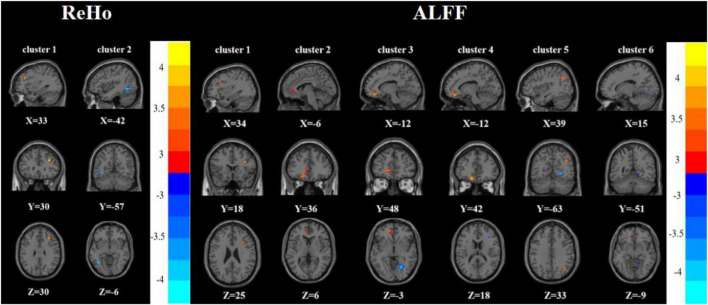
Brain regions with abnormal ReHo **(left)** and ALFF **(right)** between the RDE group and the FDE group based on a *post hoc t*-test. The color bars indicate the *T*-value.

Compared with the FDE group, the RDE group had elevated ReHo in the right inferior frontal triangular gyrus and decreased ReHo in the left inferior temporal gyrus. Meanwhile, the RDE group had elevated ALFF in the right inferior frontal triangular gyrus, left anterior cingulate gyrus, orbital part of the left middle frontal gyrus, orbital part of the left superior frontal gyrus, and right angular gyrus, but had decreased ALFF in the right lingual gyrus (Tables 2, 3 and Figure 3).

**FIGURE 4:**
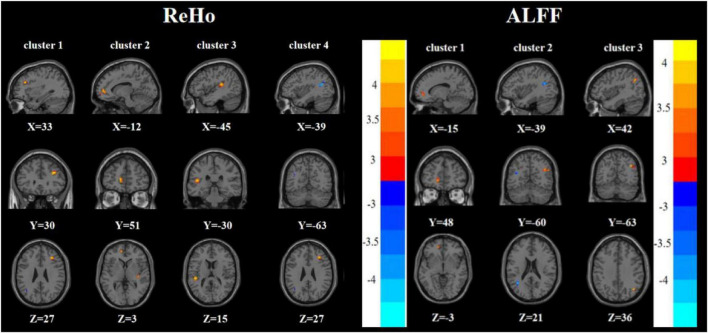
Brain regions with abnormal ReHo **(left)** and ALFF **(right)** between the RDE group and the HC group based on a *post hoc t*-test. The color bars indicate the *T*-value.

Compared with the HC group, the RDE group had elevated ReHo in the right inferior frontal triangular gyrus, left anterior cingulate gyrus, and left superior temporal gyrus, but had reduced ReHo in the left angular gyrus. Meanwhile, the RDE group had elevated ALFF in the left anterior cingulate gyrus and right angular gyrus, but had decreased ALFF in the left angular gyrus (Tables 2, 3 and Figure 4).

**FIGURE 5:**
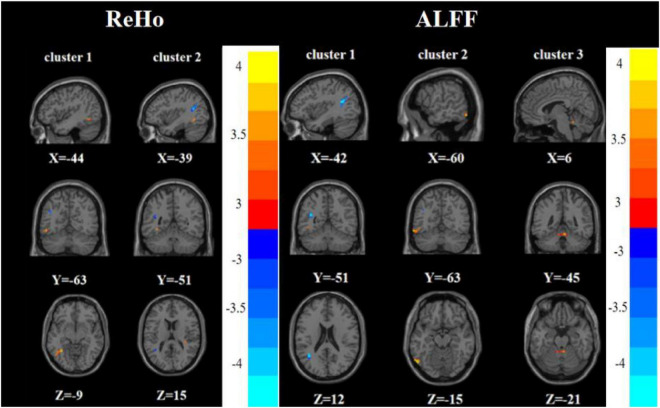
Brain regions with abnormal ReHo **(left)** and ALFF **(right)** between the FDE group and the HC group based on a *post hoc t*-test. The color bars indicate the *T*-value.

Compared with the HC group, the FDE group had elevated ReHo in the left inferior temporal gyrus and decreased ReHo in the left middle temporal gyrus/left angular gyrus. Meanwhile, the FDE group had elevated ALFF in the left inferior temporal gyrus and Vermis_3, but had decreased ALFF in the left middle temporal gyrus/left angular gyrus (Tables 2, 3 and Figure 5).

### Significant Correlation Between Functional Image and Clinical Feature

**FIGURE 6:**
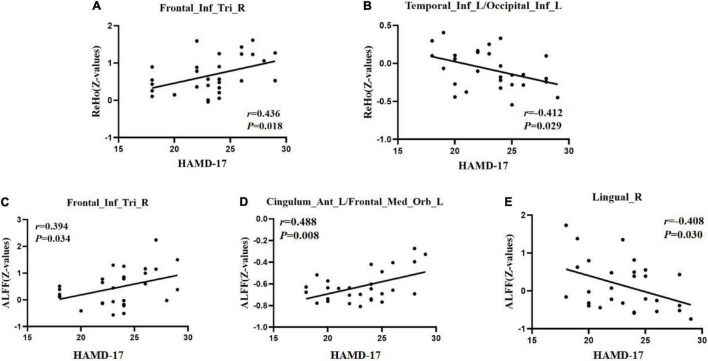
Positive correlation between the ReHo/ALFF values of abnormal brain regions and the HAMD-17 scores. **(A)** ReHo values in the recurrent depressive episode (RDE) group. **(C)** ALFF values in the RDE group. **(D)** ALFF values in the first depressive episode (FDE) group. Negative correlation between the ReHo/ALFF values of abnormal brain regions and the HAMD-17 scores. **(B)** ReHo values in the FDE group. **(E)** ALFF values in the FDE group. Frontal_Inf_Tri_R, right inferior frontal triangular gyrus; Cingulum_Ant_L/Frontal_Med_Orb_L, left anterior cingulate cortex/orbital part of the left middle frontal gyrus; Temporal_Inf_L/Occipital_Inf_L, left inferior temporal gyrus/left inferior occipital gyrus; Lingual_R, right lingual gyrus; ReHo, regional homogeneity; ALFF, amplitude of low-frequency fluctuations; HAMD-17, 17-item Hamilton Rating Scale for Depression.

To test the correlation between areas of abnormal brain activity and the severity of clinical depressive symptoms, we further performed Pearson correlation analysis. We found a positive correlation between the ReHo/ALFF values of the right inferior frontal triangular gyrus and the HAMD-17 scores in the RDE group (*r* = 0.436, *P* = 0.018; *r* = 0.394, *P* = 0.034). Meanwhile, we observed a positive correlation between the ReHo values in the left anterior cingulate cortex/orbital part of the left middle frontal gyrus and the HAMD-17 scores in the FDE group (*r* = 0.488, *P* = 0.008). Furthermore, we found a negative correlation between the ReHo values in the left inferior temporal gyrus/left inferior occipital gyrus and right lingual gyrus and the HAMD-17 scores in the FDE group (*r* = −0.412, *P* = 0.029; *r* = −0.408, *P* = 0.030) (Table 4 and Figure 6).

**TABLE 4:** Correlation of abnormal brain areas with clinical symptoms.

Variables	group	Brain regions	HAMD-17 score	
			
			Coefficient	*P*-value
ReHo	RDE	Right inferior frontal triangular gyrus	0.436	0.018^a^,^#^
	FDE	Left inferior temporal gyrus	−0.412	0.029^a^,^#^
		Left inferior occipital gyrus		
ALFF	RDE	Right inferior frontal triangular gyrus	0.394	0.034^a^,^#^
	FDE	Left anterior cingulate cortex	0.488	0.008^a^,^#^
		Orbital part of the left middle frontal gyrus		
	FDE	Right lingual gyrus	−0.408	0.030^a^,^#^

*^a^P-value from Pearson correlation (not corrected).*

*^#^Statistical significance.*

## Discussion

To our knowledge, this is the first study to investigate the changes in local brain function activity in patients with RDE or FDE using the ReHo and ALFF methods. The results of this study showed that the RDE and FDE groups had abnormal neural function activity in some of the same brain regions. ReHo and ALFF were more widely distributed in different brain regions and had more complex neuropathological mechanisms in the RDE group than in the FDE group. This study provides a reference for the differences in brain function activity between RDE and FDE.

We found increased ReHo and ALFF in the right inferior frontal triangular gyrus in the RDE group compared with the FDE group. The right inferior frontal triangular gyrus is located in the dorsolateral prefrontal cortex (DLPFC), an important component of executive function that is closely related to working memory, thought activity, and cognitive control (Wang et al., 2020; Brosch et al., 2021; Nejati et al., 2022). Previous studies have also shown that depressed mood and cognitive behavioral impairment in patients with depression are associated with abnormalities in executive function (Sun et al., 2018). Patients with mild impairment in the DLPFC often show symptoms of depression, such as loss of interest, poor memory, slow thinking, lack of motivation, and insomnia (Elliott, 2003; Li et al., 2021). Previous studies have found that the right inferior frontal triangle gyrus plays an important role in the early pathogenesis of MDD (Zhang et al., 2021) and that the gray matter volume of the DLPFC is reduced in patients with RDE after 6 weeks of antidepressant therapy (Li et al., 2010). Another study showed that stimulation of the right and left DLPFC with transcranial direct current stimulation was effective in reducing the risk of recurrence in patients with depression (Aparicio et al., 2019). The BOLD signals in the DLPFC have also been found to differ between RDE and FDE (Yuksel et al., 2018). This suggests that patients with RDE and those with FDE have differences in executive function. The correlation analysis in this study showed that the ReHo and ALFF values in the right inferior frontal triangular gyrus were positively correlated with the HAMD-17 scores in the RDE group, whereas no such correlation was found in the FDE group. These results suggest that the right inferior frontal triangular gyrus is an important neurobiological imaging marker for RDE and an important brain region for differentiating RDE from FDE.

We also found differences in ALFF in the left anterior cingulate gyrus and left orbitofrontal gyrus between the RDE and FDE groups. The anterior cingulate gyrus is an important component of the limbic system. It has extensive fibrous connections to many cortical and subcortical structures; is involved in the regulation of a wide range of functions, such as emotion, cognition, and motivation; and is closely associated with the onset of depression (Xiao and Zhang, 2018; Zheng et al., 2018; Rolls, 2019). Previous studies have found higher ALFF in the right ventral anterior cingulate gyrus in patients with anxious depression than in patients with rMDD and in HCs. In addition, abnormal activation of the anterior cingulate gyrus at rest in patients with MDD may be related to the failure of emotional control, which is a central factor in negative rumination and persistent self-focus in these patients (Amodio and Frith, 2006; Liu et al., 2015). Therefore, the results of this study suggest that patients with RDE show abnormal activation of the left side of the anterior cingulate gyrus. The orbitofrontal cortex is an important part of the reward network and is closely associated with emotional information and sensory stimuli (O’Doherty, 2004; Liu et al., 2017). A previous study found that patients with refractory depression, which is characterized by persistence and recurrence, had significantly higher ALFF in the orbitofrontal cortex than HCs (Liu et al., 2014). This suggests that abnormal activation of the orbitofrontal cortex is a cause of the complexity of RDE. Another study also showed that patients with FDE had reduced ALFF in the left and right orbitofrontal cortex compared with HCs, suggesting that patients with FDE have reduced regulation of the reward network (Zhang et al., 2014). Although these results were from different studies, they all support the involvement of the anterior cingulate gyrus and orbitofrontal cortex in regulating the pathophysiology of MDD. The correlation analysis in the current study showed that the ALFF values in the left anterior cingulate cortex/orbital part of the left middle frontal gyrus were positively correlated with the HAMD-17 scores in the FDE group, thus identifying an important brain region for differentiating between RDE and FDE.

In addition, we found that the RDE group had higher ALFF values in the right lingual gyrus than the FDE group. The lingual gyrus is part of the occipital lobe, and previous studies have shown that the lingual gyrus is also involved in activities related to visual memory processing and is closely associated with the development of MDD (Jung et al., 2014; Le et al., 2017; Palejwala et al., 2021). One study found that patients with rMDD had lower ALFF values in the right lingual gyrus than HCs, suggesting that reduced ALFF in the right lingual gyrus is a marker for the remission of depression (Yang et al., 2018). Another study also showed that patients with FDE had lower ALFF values in the right lingual gyrus than HCs (Wang et al., 2012). Our correlation analysis showed that the ALFF values in the right lingual gyrus were negatively correlated with the HAMD-17 scores in the FDE group. This suggests that the right lingual gyrus can be used as a status marker for FDE.

Meanwhile, we found that the ALFF in the right angular gyrus was higher in the RDE group than in the FDE group. A previous study reported that the right angular gyrus is associated with the self-localization function in humans and is potentially associated with psychiatric disorders, and that an abnormality in this function affects both sensory and perceptual functions, with a causal relationship between them (de Boer et al., 2020). This may also be informative in elucidating the differences in neuropathological mechanisms between RDE and FDE. The inferior temporal gyrus is involved in functions such as social cognition, emotional stimulus processing, self-referential processing, and semantic processing and is closely associated with MDD development (Chao et al., 1999; Cabeza and Nyberg, 2000; Herath et al., 2001; Hu et al., 2017; Kocsis et al., 2021). Previous studies have found that ReHo is significantly higher in the left middle temporal gyrus in patients with FDE than in those with rMDD, suggesting that enhanced metabolism in the left middle temporal gyrus may be one of the pathogenic mechanisms of FDE (Yang et al., 2018). Therefore, we suggest that differences exist between RDE and FDE in terms of abnormalities in the frontal, temporal, parietal, and occipital lobes, particularly in the right inferior frontal triangular gyrus of the frontal lobe.

Interestingly, we also found that the RDE and FDE groups had lower ReHo and ALFF in the left angular gyrus than the HC group. The angular gyrus is located in the posterior part of the inferior parietal lobe and is mainly involved in human semantic and numerical processing, memory retrieval, spatial cognition, word reading and comprehension, reasoning, and social cognition, with the left angular gyrus playing a more important role in situational simulation and memory (Seghier, 2013; Thakral et al., 2017; Ramanan and Bellana, 2019). The angular gyrus is also an important part of the default mode network (Raichle et al., 2001; Raichle, 2015). Previous studies have found differences in BOLD signals in the angular gyrus between patients with RDE or FDE and HCs, consistent with the present study (Yuksel et al., 2018). Other studies have also reported that the ReHo and ALFF values in the left angular gyrus were lower in patients with MDD than in HCs (Wang et al., 2012; Liu et al., 2021). Abnormalities in angular gyrus function can lead to cognitive impairment, a common clinical manifestation of MDD (Lee et al., 2012). Therefore, this suggests that (1) both RDE and FDE are characterized by default mode network dysfunction and (2) ReHo and ALFF abnormalities in the left angular gyrus are important markers for differentiating patients with MDD from HCs.

Some limitations of this study should be considered. First, the patients with RDE enrolled in this study might have still been affected by an underlying antidepressant action despite having stopped medication for 4 weeks. Second, the number of recurrences in the RDE group was inconsistent, and a first-recurrence study of patients with RDE seem to have greater research value. Third, this study focused on only one scale (HAMD-17). To enhance the scientific value of this study, more scales need to be used in the future to focus on the detailed correlation of cognitive, somatic, anxiety, and insomnia symptoms with RDE and FDE. Finally, this study did not find a difference between RDE and FDE in depressive symptoms, which may be related to the specificity and small size of the study population. Further studies with a larger sample size are needed to confirm or refute the findings of this study.

## Conclusion

This study used ReHo and ALFF, which are indices based on the rs-fMRI technique, to preliminarily analyze the differences between RDE and FDE in terms of neural activity in different brain regions. The RDE and FDE groups showed abnormal changes in neural function activity in some of the same brain regions, with ReHo and ALFF being more widely distributed in different brain regions and the neuropathological mechanisms being more complex in the RDE group than in the FDE group, particularly in the right inferior frontal triangular gyrus of the frontal lobe.

## Data Availability Statement

The raw data supporting the conclusions of this article will be made available by the authors, without undue reservation.

## Ethics Statement

The experimental protocol was approved by the Ethics Committee of Guang’anmen Hospital, Chinese Academy of Traditional Chinese Medicine (No. 2017-021-SQ), Trial registration: China Clinical Trials Registry, chiCTR1800014277. The patients/participants provided their written informed consent to participate in this study.

## Author Contributions

JF conceived and designed this experiment. JS wrote and revised the manuscript and participated in the collection of cases and statistical analysis of the data. LC drew diagrams and made statistical analysis of data and revised the manuscript. ZD analyzed the data and revised the manuscript. JH, DG, YM, ZW, CG, and YL participated in data analysis, case collection, and manuscript writing. YH, LZ, and JC performed fMRI on the patient. FX, XH, XX, JT, and XY involved in case collection and symptom assessment of patients. All authors contributed to the article and approved the submitted version.

## Conflict of Interest

The authors declare that the research was conducted in the absence of any commercial or financial relationships that could be construed as a potential conflict of interest.

## Publisher’s Note

All claims expressed in this article are solely those of the authors and do not necessarily represent those of their affiliated organizations, or those of the publisher, the editors and the reviewers. Any product that may be evaluated in this article, or claim that may be made by its manufacturer, is not guaranteed or endorsed by the publisher.

## Footnotes

1
http://www.rfmri.org/DPARSF
